# Fiscal policy, inequality, and poverty in Iran: assessing the impact and effectiveness of taxes and transfers

**DOI:** 10.1080/17938120.2019.1583510

**Published:** 2019-03-08

**Authors:** Ali Enami, Nora Lustig, Alireza Taqdiri

**Affiliations:** a Cornerstone Research, Two Embarcadero Center, 20th Floor, San Francisco, USA; b Department of Economics and CEQ Institute, Tulane University, New Orleans, LA, USA; c Brookings Institution, Center for Global Development, and Inter-American Dialogue; d Department of Economics, Concordia University, Montreal, Canada

**Keywords:** Incidence analysis, marginal contribution, effectiveness, energy subsidy reform, Iran, D31, H22, I38

## Abstract

Using the Iranian Household Expenditure and Income Survey for 2011/12, we estimate the impact and effectiveness of various components of Iran’s fiscal system on reducing inequality and poverty. We utilize the marginal contribution analysis to determine the impact of each component, and we introduce newly developed indicators of effectiveness to calculate how well various taxes and transfers are operating to reduce inequality and poverty. We find that the fiscal system reduces the poverty-head-count-ratio by 10.5 percentage points and inequality by 0.0854 Gini points. Transfers are generally more effective in reducing inequality than taxes while taxes are especially effective in raising revenue without causing poverty to rise. Although transfers are not targeted toward the poor, they reduce poverty significantly. The main driver is the Targeted Subsidy Program (TSP), and we show through simulations that the poverty reducing impact of TSP could be enhanced if resources were more targeted to the bottom deciles.

## I. Introduction

In December 2010, Iran’s government replaced its energy and bread subsidies with a lump-sum cash transfer known as the Targeted Subsidy Program (TSP) (Guillaume, Farzin, & Zytek, 2011).^1^ The removal of (mainly, energy) subsidies resulted in an increase of about 21% in prices. Had the reforms stopped there, the poor would have been hurt. At the same time, to garner political support for the reform, the nonpoor had to be awarded a certain degree of protection from the rise in prices of previously subsidized goods. Hence the rationale for a universal cash transfer rather than a targeted one (Guillaume et al., 2011; Mostafavi-Dehzooei & Salehi-Isfahani, 2017; Salehi-Isfahani, Stucki, & Deutschmann, 2015).^2^

This paper analyzes to what extent the TSP reduces poverty under the new scenario of higher prices. By using information from a household survey collected several months after the reform, one can assume that the ensuing increase in prices due to this reform is already embedded in the survey. Likewise, one can assume that the survey has captured the adjustment in consumption patterns that the reform might have induced. To answer this question, we measure the impact of TSP on inequality and poverty by comparing it to a counter factual world in which the reform did not include the cash transfer component.^3^ Although Salehi-Isfahani et al. (2015) find that TSP reduced inequality and poverty when compared to the hypothetical case of households receiving neither TSP nor a consumption subsidy, they only looked at the impact of this reform three months into its implementation.^4^ Moreover, they relied on indirect methods to determine who received TSP because the survey they used did not include an explicit question about this program. After their paper was published, Iran released the Household Expenditure and Income Survey (HEIS) for 2011/12 (1390 by the Iranian calendar) which did include specific questions on how much the household received in TSP transfers and how many people in the household received them. Therefore, we can estimate the impact of TSP transfers with actual data on benefits, rather than relying on the indirect method. This is the first contribution of this paper.

As Salehi-Isfahani et al. (2015) indicate, the reform did increase the fiscally-induced reduction in inequality and poverty from the start, but it did not reduce the government’s fiscal burden. Spending on TSP exceeded the additional revenue generated from the increase in the prices of previously subsidized energy goods in large part because energy consumption was lower without the subsidies, but also because of the reduction in international oil prices (Salehi-Isfahani et al., 2015). In the first eighteen months of this reform, spending on TSP was almost twice the amount of the increase in government revenue that resulted from eliminating the energy subsidies (Iranian Labour News Agency, 2013).^5^ To address this problem, the Iranian government decided in 2014 to switch from a universal cash transfer to one that prevented the top 20% of the population from receiving TSP. The government called this change the ‘Second Phase’ of the subsidy reform, but it was not able to properly implement it to this day due to the pressure from the public. The government has only been able to remove the cash transfer from a very small percentage of the rich population. Here, we analyze what would have been the impact on inequality and poverty, and the fiscal resources saved, if the design of the transfer had excluded the top 20% from the start. In a way, one can consider the extra budgetary outlays as an estimate of the fiscal cost associated with making the reform politically palatable to the population as a whole. This is the second contribution of this paper.

While eliminating the cash transfer from the rich households can reduce the financial burden of the program without hurting the poor, its effectiveness in reducing poverty can increase if it is distributed in a more targeted way. Therefore, a third contribution of this paper is an assessment of the extent to which making the TSP more targeted would be more effective in protecting the poor and would reduce fiscal outlays. Specifically, we analyze how much the contribution of this program to reducing inequality and poverty, and TSP’s overall effectiveness, would change if in addition to the elimination of the cash transfer from the top two decile, deciles VII and VIII were also no longer eligible and the resulting savings from the latter two deciles were transferred to the remaining income deciles (policy simulation 1) or to the bottom 30% (policy simulation 2).^6^

To estimate the impact of both the universal and ‘Second Phase’ of TSP, as well as policy simulations 1 and 2, we rely on standard fiscal incidence analysis as described in Lustig (2018). Fiscal incidence analysis is used to assess the distributional impacts of a country’s taxes and transfers.^7^ Essentially, it consists of allocating taxes (particularly the personal income tax and consumption taxes) and public spending (particularly social spending) to households or individuals in order to compare incomes before taxes and transfers to incomes after taxes and transfers. Transfers include: direct cash transfers; in-kind benefits, such as free government education and health care services, and consumption subsidies, including food, electricity, and fuel subsidies. Our analysis includes: personal income taxes and contributions to health insurance and social security, Social Assistance, TSP and other direct transfers, sales taxes, and in-kind transfers in education and health (net of user fees). Because standard fiscal incidence analysis, such as the one applied here, ignores behavioral responses and general equilibrium effects, our exercise estimates the direct effects of subsidies (and their removal) only. Thus, it is a useful first-order approximation of the effects of this fiscal policy. Furthermore, this analysis is one of the very few available for Iran, especially since its sweeping energy subsidy reform.

To measure the contribution of taxes and transfers to fiscally-induced changes in inequality and poverty, we use the marginal contribution approach (Enami, Lustig, & Aranda, 2018; Lambert, 2001). By this method, the contribution of a tax or a transfer to a change in inequality is measured by comparing the existing fiscal system to a counter-factual that excludes the tax (or transfer) of interest.^8^ This approach is superior to using progressivity indicators (such as the Kakwani index) for determining whether a tax (or transfer) is inequality-increasing (or decreasing). This is because standard progressivity indicators can yield the wrong prediction, in terms of the impact of a particular intervention, when the number of fiscal instruments is greater than one. When a fiscal system is composed of multiple taxes and transfers, a progressive tax (or transfer) can actually increase inequality and a regressive tax (transfer) can reduce inequality.^9^

While a specific tax (transfer) can have a large effect on reducing inequality (or poverty), one key concern for economists and policymakers is to determine whether that tax (transfer) is effective. In this paper, we follow Fellman, Jäntti, and Lambert (1999) and Enami (2018b), and define effectiveness by comparing how close the actual marginal contribution of a tax (transfer) comes to achieving its maximum potential. We show, for example, that despite its relatively large effect on poverty and inequality, TSP is relatively less effective compared to some other components of the fiscal system in Iran. This finding highlights the importance of better targeting of cash subsidies, and motivates our policy simulations.

Our results show that the fiscal system in Iran (including direct and indirect taxes, direct transfers, and in-kind transfers for education and health) reduces the Gini coefficient by 0.0854 points, or 20%, compared to the Market Income Gini. Excluding the in-kind transfers for education and health, the reduction equals 0.0574 Gini points, or 13% of the Market Income Gini. Moreover, Iran’s fiscal system is quite powerful in reducing poverty. The headcount ratio falls from about 21% to 11%.^10^^,^^11^

We also find that taxes are very effective in raising revenue without increasing poverty, and are moderately effective in reducing inequality. In contrast, because transfers are universal and not targeted to poor households, they realize only about 16% of their potential to reduce poverty. In terms of inequality, transfers are more similar to taxes: they moderately realize their potential. The ‘Social Assistance’ program leads other interventions, with a realized power of about 40% to 42%. Among taxes, only the Income Tax displays an effectiveness of this magnitude (about 34% to 36%).

Based on the size of its marginal contribution, TSP has the greatest impact in reducing inequality and poverty. TSP actually reduced inequality by about 0.0552 Gini points. Without TSP, the poverty headcount ratio would have been about 22% rather than 12%. This reduction in poverty comes mainly from the large effect of this program in rural areas. Without it, the headcount ratio in rural areas would have been about 44%, not the observed 23% (while the headcount ratio in urban areas would have been 13%, not the observed 6%).^12^ However, TSP’s ‘success’ is mainly due to its size. Because it is basically universal, it is not effective in the sense that much more could be achieved in terms of reducing inequality and poverty if the resources were better targeted to the poor.

Given the importance of the TSP, we also evaluate two alternative scenarios of allocating its resources. We show that removing the subsidy from deciles VII and VIII, and allocating the additional savings to the bottom 60% (policy simulation 1), or just to the bottom 30% (policy simulation 2), would significantly reduce inequality and poverty. This is mainly because the program is already very successful in reaching the low-income groups, especially in rural areas.

The rest of this paper is organized as follows: Section II briefly reviews Iran’s fiscal system and lists the programs that are included in the analysis. It also explains the method and assumptions used to construct items not directly observed in the household survey. Section III discusses the data and methodology used in this paper, specifically the marginal contribution approach to calculating the effect of different taxes and transfers on reducing (increasing) inequality and poverty. We also describe the effectiveness indicators used in our analysis. Section IV presents the results of our inequality and poverty analysis. We pay special attention to the Target Subsidy Program because of its significant role in reducing inequality and poverty. Finally, Section V concludes and presents policy recommendations for moving forward in managing the TSP in Iran.

## II. Overview of Iran’s fiscal system and the taxes and transfers included in this analysis

**Figure 1.**
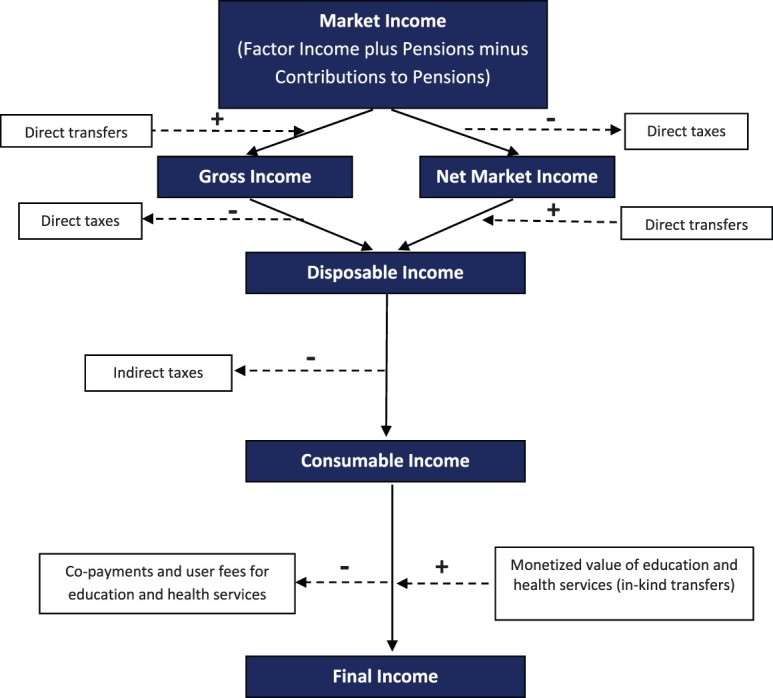
A framework to define income concepts and combine fiscal interventions. Source: Lustig (2018) with some adaptation. Note: Core Income Concepts in dark blue background, Fiscal Interventions in white background.

Iran’s fiscal system is composed of taxes, transfers, subsidies, and pensions which are briefly described in appendix A. In this Appendix, we indicate which components are included in the analysis and what assumptions are used to construct their values if they are not directly observed in the household survey. Note that the information in this Appendix closely relates to Figure 1 and Section III on methodology.

To provide some context here, Table 1 presents a summary of the revenue sources and expenditure areas of Iran’s budget (2011/12). Total revenues and spending are roughly the same: about 164 billion dollars, which is about 27% of GDP. The main source of revenue is natural resources (mainly oil), followed by capital and financial assets (55.23% of budget), and finally by tax revenues (24.0% of budget). Government expenditures are divided equally into social expenditures and all other types of expenditures (e.g. defense). Education, social protection, TSP, and health expenditures are the main categories of social expenditures with 16.58%, 11.84%, 10.91%, and 9.24% of the budget allocated to them respectively. Table 1 also shows the categories that were included in the analysis.

**Table 1.** Iranian government revenues and expenditures (1390 Iranian calendar, equivalent to 2011–12).

**Panel A. Government revenues**			
Categories	% of total revenue	% of GDP	Included in analysis
Total Revenues	100%	**27.00%**	
Tax revenues	24.07%	6.50%	
Direct taxes, of which:	14.21%	3.84%	
Personal Income Tax	3.14%	0.85%	Yes
Corporate Income Tax	10.26%	2.77%	No
Wealth Tax	0.81%	0.22%	No
Indirect Taxes	9.86%	2.66%	Yes
Non-tax revenues	75.93%	20.50%	
Sales of natural resources, capital, and financial assets	55.23%	14.91%	No
Other Revenues	20.70%	5.59%	No

**Panel B. Government expenditures**			
Categories	% of total expenditure	% of GDP	Included in analysis
Total expenditure	100%	27.00%	
Social spending	50.68%	13.69%	
Targeted Subsidy Program	10.91%	2.95%	Yes
Social protection	11.84%	3.20%	
Social assistance, of which:	3.85%	1.04%	
Assistance to the Low-Income Families and Orphans	1.59%	0.43%	Yes
Assistance to the Families of Martyrs and wounded soldiers.	2.23%	0.60%	Yes
Other	0.03%	0.01%	Yes
Social security, of which:	7.99%	2.16%	
Retirement Pensions: Civilians	4.49%	1.21%	Yes
Retirement Pensions: Armed Forces	3.50%	0.95%	Yes
Education, of which:	16.58%	4.48%	
12-K (Primary and Secondary)	7.79%	2.10%	Yes
Adult Literacy	0.14%	0.04%	No
Tertiary	7.89%	2.13%	Yes
Other	0.76%	0.20%	No
Health	9.24%	2.50%	Yes
Housing (urban and rural)	2.12%	0.57%	No
Other expenditures	49.32%	13.32%	No

Source: Own calculations using Adlband (2011) and SCI (2015).

Note: The total revenues and expenditures are equal to each other and equal to 1,697,255 billion Rials (about 163.76 billion dollars). The raw data is from Iran’s budget in which total revenues and expenditures are equal, but that is due to the elements such as borrowing from the public and banks as well as the sales of public firms which close the deficit gap. The GDP of Iran for this period is 6,285,255 billion Rials (about 606.45 billion dollars).

## III. Methodology and data

Fiscal incidence analysis begins with constructing basic income concepts. Figure 1 presents the generally defined income concepts. In the Methodological Appendix (Appendix B), we describe in greater detail how these income concepts are constructed for Iran. In broad terms, we begin with Market Income,^13^ then subtract direct taxes and add cash transfers to obtain Disposable Income. Next, we subtract indirect taxes to generate Consumable Income. Because TSP replaced consumption subsidies, there are no consumption subsidies in our model. Finally, we add the monetized value (at average government cost) of In-kind transfers (i.e. health and education), net of user fees, to obtain Final Income.

This study relies on the concept of marginal contribution to estimate the contribution of taxes and transfers to reducing inequality and poverty. Theoretically, marginal contribution analysis asks what the distribution of income would have been in the absence of a tax^14^ (or transfer), defining the difference between this counter factual and the actual distribution of income as the marginal contribution of that tax (or transfer). This is shown in the equation below:

$${\mathrm{MC}}_{T(\mathrm{or}\;B)}^{\mathrm{End}\;\mathrm{income}}=\mathrm{Inde}x_{\mathrm{End}\;\mathrm{income}\setminus T(\mathrm{or}\;B)}-\mathrm{Inde}x_{\mathrm{End}\;\mathrm{income}};$$

where $\;{\mathrm{MC}}_{T\;(or\;B)}^{\mathrm{End}\;\mathrm{income}}$ is the marginal contribution of tax or transfer to the inequality or poverty index of an ‘end income’ concept (such as the disposable income). $\mathrm{Inde}x_{\mathrm{End}\;\mathrm{income}\setminus T(\mathrm{or}\;B)}$ is the value of that index for the same end income concept but when *T* (or *B*) is excluded. Similarly, $\mathrm{Inde}x_{\mathrm{End}\;\mathrm{income}}$ is the value of that index when *T* (or *B*) is included. For example, the marginal contribution of direct taxes to the redistributive effect from market income to disposable income equals the difference between the Gini of disposable income including the direct taxes and the Gini coefficient of disposable income alone. In this paper, we focus on the first order effects of removing a tax or transfer and therefore ignore the behavioral responses. As is clear from the equation above, the order in which other fiscal interventions are added has no effect on the value of the $\mathrm{Inde}x_{\mathrm{End}\;\mathrm{income\setminus}T(\mathrm{or}\;B)}$.

One important feature of the marginal contribution approach is that it does not rely on the order in which other taxes and transfers (besides the tax or transfer of interest) are incorporated into the calculation. However, there is no guarantee that the sum of the marginal contributions of all components of a fiscal system is equal to the overall redistributive effect. This mathematical constraint has no implication for policy makers. Policy questions are all about changing the characteristics of a particular tax or transfer, adding a tax or transfer, or eliminating a tax or transfer, and how such changes potentially would affect the redistributive and poverty indicators. Only the marginal contribution approach provides the correct answer to these questions by comparing the fiscal system before and after a tax, transfer, or particular reform.

We use ‘Impact and Spending Effectiveness Indicators’ to evaluate how well taxes and transfers reduce inequality. In order to assess the effectiveness of taxes, transfers, or changes in them, we rely on the notion of ‘optimal tax (transfer)’ (Fellman et al., 1999),^15^ using the indicators proposed in Enami (2018b) which are described below.

Mathematically, a given amount of taxes (or transfers) can be collected (allocated) in such a way to maximize the impact on inequality (or poverty) reduction. For example, in the case of the Gini coefficient, the maximum effect is obtained by collecting taxes from the richest individual until his/her income becomes equal to the second richest, then taxing both of them until their income becomes equal to the third richest person, and to continue this process until all of the tax has been collected. This procedure maximizes the reduction in Gini while keeping the size of taxes constant. An ‘optimal’ transfer would follow a similar procedure, but start with the poorest individual and move him/her up in the income distribution. This indicator is defined as follows:

$${\mathrm{Inequality}\;\;\mathrm{Impact}\;\;\mathrm{Effectiveness}}_{T(\mathrm{or}\;B)}^{\mathrm{End}\;\mathrm{income}}=\frac{{\mathrm{MC}}_{T(\mathrm{or}\;B)}^{\mathrm{End}\;\mathrm{income}}}{\mathrm{MC}{{}_{T(\mathrm{or}\;B)}^{\mathrm{End}\;\mathrm{income}}}^{*}}\;;$$

where ${\mathrm{MC}}_{T\;(\mathrm{or}\;B)}^{\mathrm{End}\;\mathrm{income}*}$ is the maximum possible ${\mathrm{MC}}_{T\;(\mathrm{or}\;B)}^{\mathrm{End}\;\mathrm{income}}$ if the same amount of Tax (or Benefit) is levied on (distributed among) individuals optimally. The ‘end income’ in our analysis can refer to one of three income concepts: Disposable Income, Consumable Income, and Final Income (defined in Figure 1). The value of this Inequality Impact Effectiveness indicator lies between −1 and +1 (the higher the indicator, the more effective).

Alternatively, one can keep the change in inequality constant and estimate the minimum size of a tax or a transfer that would be required to achieve the same marginal contribution. This reduction in the size of a tax or transfer is obtained through the same optimal redistribution process described above. This indicator is defined as follows:

$${\mathrm{Inequality}\;\;\mathrm{Spending}\;\;\mathrm{Effectiveness}}_{T(\mathrm{or}\;B)}^{\mathrm{End}\;\mathrm{income}}=\frac{T^{*}(\mathrm{or}\;B^{*})}{T(or\;B)};$$

where $T^{*}(\mathrm{or}\;\;B^{*})$ is the minimum amount of T (or B) that is needed to create the same ${\mathrm{MC}}_{T\;(\mathrm{or}\;B)}^{\mathrm{End}\;\mathrm{income}}$ if the tax or transfer were optimally redistributed. Note that the Spending Effectiveness Indicator is only calculated for taxes and transfers with a positive ${\mathrm{MC}}_{T\;(\mathrm{or}\;B)}^{\mathrm{End}\;\mathrm{income}}$ because it is meaningless to calculate the optimum size of a tax or transfer that increases inequality. As a result, the value of this indicator lies between 0 and 1 (the higher the indicator, the more effective).

We use Impact and Spending Effectiveness Indicators to evaluate the performance of taxes and transfers in reducing inequality. Although we have shown results using the Gini coefficient, the indicators can be calculated with any other inequality measure.

To evaluate how taxes and transfers reduce poverty, we need a different index. Higgins and Lustig (2016) show that fiscal policies usually create both fiscal gain to the poor (FGP) and fiscal impoverishment (FI). Thus, one should differentiate between the two effects. Therefore, we use FI-FGP effectiveness indicators to account for these two effects. Although FI-FGP indicators are conceptually similar to our Impact Effectiveness indicators, one should not compare the FI-FGP effectiveness of taxes to transfers. Taxes can only hurt the poor (i.e. by increasing FI), while transfers can only benefit the poor (i.e. by increasing FGP). The FI-FGP indicators are defined so that the higher their value, the better a tax or transfer is. But the interpretations are different: the higher the value of the FI-FGP indicator for a tax, the more successful that tax is in raising revenue without increasing poverty; the higher the value of this indicator is for a transfer, the more successful it is in reducing poverty.

The FI-FGP indicators are calculated as follows:

$$\mathrm{FI\_FG}P_{T}=\frac{T-\mathrm{FI}\_{\mathrm{MC}}_{T}^{\mathrm{End}\;\mathrm{income}}}{T};$$

$$\mathrm{FI\_FG}P_{B}=\frac{{\mathrm{FGP\_MC}}_{B}^{\mathrm{End}\;\mathrm{income}}}{B};$$

$$\mathrm{FI\_FG}P_{\mathrm{Total}\;\mathrm{system}}=\left[\left(\frac{B}{T+B}\right)\left(\frac{{\mathrm{FGP\_MC}}_{B}^{\mathrm{End}\;\mathrm{income}}}{B}\right)\right]+\left[\left(\frac{T}{T+B}\right)\left(\frac{T-{\mathrm{FI\_MC}}_{T}^{\mathrm{End}\;\mathrm{income}}}{T}\right)\right]$$

${\mathrm{FI\_MC}}_{T}^{\mathrm{End}\;\mathrm{income}}$ is the marginal contribution of tax T to the Fiscal Impoverishment (FI) index of the end income of interest and ${\mathrm{FGP\_MC}}_{B}^{\mathrm{End}\;\mathrm{income}}$ is the marginal contribution of transfer B to the Fiscal Gain to Poor (FGP) index of the end income of interest. The FI indicator measures how much poor individuals become worse off and non-poor become poor as a result of a tax. The FGP indicator measures how much poor individuals are made better off as a result of a transfer. Following Higgins and Lustig (2016), the change in the poverty gap is the index used to calculate the FI-FGP indicators.

All FI_FGT indicators vary between zero and one (the higher the indicator, the better). However, one cannot compare the effectiveness of taxes to transfers because taxes can only increase poverty. So, their effectiveness is calculated with respect to how much they do not increase poverty while raising revenue. On the other hand, transfers can only reduce poverty, so their effectiveness is calculated with respect to their performance in reducing poverty. The total fiscal system, which is the combination of all taxes and transfers, can increase or decrease poverty. Therefore, it should be only compared to alternative fiscal systems that have both taxes and transfers.

The main data base for this study is the Iranian Household Expenditure and Income Survey (HEIS) for the calendar year 1390 (2011–12).^16^ The Statistical Center of Iran conducts this survey every year, and its sample represents all rural and urban areas of Iran. In the survey year that we use, there are 18,727 urban and 19,786 rural households in the sample. These households represent about 56.4 million urban and 23.1 million rural individuals. For each of the households in the sample, we follow Figure 1 and construct the core income concepts as well as income components (i.e. taxes and transfers) as described in Table B1 in the Methodological Appendix. As mentioned earlier, the marginal contribution technique used in this paper is not sensitive to the order of adding taxes and transfers.

Table 2 shows the distribution of individuals and households based on their income group and the average household size in each income group. About 21% of the population live in poverty and 41% are economically vulnerable. Together, about 62% of Iranians are considered low-income. The middle class is also large and includes about 37% of the population. The remaining 1% belong to the high-income group.

**Table 2.** Distribution of individuals and households according to socio-economic group.

In Daily US 2005 PPP	Socio-Economic Group	Number of individuals (% share)	Number of households (% share)	Average size of household
0–1.25	Ultra Poor	2,875,462 (3.62%)	729,004 (3.45%)	3.9
1.25–2.5	Extreme Poor	5,284,959 (6.65%)	1,305,675 (6.17%)	4.0
2.5–4	Moderate Poor	8,586,729 (10.80%)	1,930,893 (9.13%)	4.4
4–10	Vulnerable	32,281,101 (40.60%)	7,810,339 (36.91%)	4.1
10–50	Middle Class	29,755,312 (37.42%)	9,026,572 (42.66%)	3.3
50 or more	High Income Class	728,130 (0.92%)	356,549 (1.69%)	2.0
Total		79,511,694	21,159,033	3.8

Source: Own calculations using the Iranian household survey (1390 Iranian calendar, equivalent to 2011–12).

Note: The total population slightly exceeds the actual population for this year due to the application of survey weights. Socio-Economic group is determined according to the ‘Market Income’. PPP stands for Purchasing Power Parity. In calculating PPP values, we use the 2005 round of ICP (International Comparison Program) as reported in the World Development Indicators (WDI) published by the World Bank. To change monetary values from the year of survey to 2005, we use the CPI index from the WDI.

## IV. Results

In this section, we first analyze each component of the fiscal system and evaluate its marginal contribution to reducing inequality and poverty, as well as its effectiveness in doing so. then, we focus on the ‘Targeted Subsidy Program,’ and evaluate how much it would contribute to the change in poverty and inequality (in terms of marginal contribution) and its effectiveness in different policy scenarios. It is important to note that throughout our analysis, all income values are in per capita terms and poverty lines are appropriately adjusted to reflect the per capita nature of the data.

### IV.A. Contribution of fiscal interventions to changes in inequality and poverty

Table 3 shows the progressivity of each income component of the fiscal system, as well as its marginal contribution to reducing (or increasing) inequality for three of the main income concepts (i.e. Disposable, Consumable, and Final Incomes). The interpretation of marginal contributions is as follows: how much the Gini of an income concept would have been higher (or lower) if a specific income component (i.e. a tax or transfer) were removed from the fiscal system. Positive values mean that the Gini would have been higher; therefore, removing that component increases inequality. Put differently, positive values for the marginal contribution mean that an income component has a positive effect in increasing equality (or reducing inequality). Among all the income components, Semi-cash Transfers (Food), indirect taxes (i.e. Sales Taxes), and Health User-fees have a negative effect on equality. As expected, direct transfers make the highest marginal contribution to reducing inequality in all three income concepts. However, the main contribution comes from the Targeted Subsidy Program with a marginal contribution of about 0.05 Gini points. This is in line with findings of Cockburn, Robichaud, and Tiberti (2018), that utilize ex-ante simulations of energy subsidy reform proposals in Egypt and Jordan (two countries that are also in the Middle East region) to show that using cash transfers to reallocate part of the freed-up resources would have a significant effect on reducing poverty in these two countries.

**Table 3.** Marginal contribution of taxes and transfers to inequality.

Fiscal Intervention		Progressivity (Kakwani Index)	Marginal contribution to the Gini index of:		
			Disposable Income (0.3686)	Consumable Income (0.3712)	Final Income (0.3432)
Direct Taxes and Contributions	Income Tax	0.2274	0.0018	0.0018	0.0019
	Employee contributions to the health insurance	0.0002	0.0003	0.0002	0.0004
	Employer contributions to the health insurance	0.0455	0.0008	0.0007	0.0009
	Total Direct Taxes and Contributions	0.0855	0.0029	0.0028	0.0032
Direct Transfers	Targeted Subsidy Program	0.4164	0.0527	0.0552	0.0465
	Social Assistance	0.8205	0.0043	0.0045	0.0040
	Semi-cash Transfers (Food)	0.3018	<0.0000	<0.0000	<0.0000
	Total Direct Transfers	0.4384	0.0583	0.0611	0.0516
Indirect Taxes (Sales Taxes)		−0.1363	–	−0.0026	−0.0025
In-kind Transfers	Education Transfers	0.3485	–	–	0.0226
	Education User-fees	0.0682	–	–	0.0018
	Health Transfers	0.4171	–	–	0.0177
	Health User-fees	0.1611	–	–	−0.0075
	Total In-kind Transfers	0.5886	–	–	0.0290

Source: Own calculations using the Iranian household survey (1390 Iranian calendar, equivalent to 2011–12).

Note: The Kakwani index is calculated with respect to the ‘Market Income’.

Table 3 also reveals two examples of a phenomenon known as the Lambert Conundrum (Enami et al., 2018). The commonly used rule of thumb regarding the effect of a tax or transfer on reducing inequality states that a progressive tax or transfer (as measured by the Kakwani index) reduces inequality and a regressive tax or transfer increases it. However, this rule is not always correct, because adding a regressive tax (or transfer) can result in higher equality, or adding a progressive tax (or transfer) can increase inequality. In Iran’s case, the Semi-Cash Transfer (Food) and Health User-fees are progressive (have a positive Kakwani index) but their marginal contributions to the inequality of Final Income (and other Income concepts for the Semi-Cash Transfer) are negative. In other words, removing these progressive interventions would result in lower (instead of higher) inequality over the whole income distribution.^17^

Table 4 does the same marginal contribution analysis for the poverty headcount ratio. In this table, positive values have a positive connotation, similar to that of the previous table. In other words, a transfer with a positive marginal contribution would reduce poverty; if it is removed from the fiscal system, the result would be an increase in the poverty headcount ratio equal to the size of the marginal contribution. As expected, taxes always can do harm, i.e. increase poverty, but they are not a concern in the case of Iran except for the Sales Taxes. With respect to Consumable Income and $4PPP poverty line, Direct Taxes increase the poverty headcount ratio by about 0.2 percentage points and Sales Taxes increase it by about 1.2 percentage points. On the other hand, direct transfers reduce this poverty index by about 12.8 percentage points. Most of this effect is due to the Targeted Subsidy Program, which reduces poverty by about 11.9 percentage points. To put this value in context, note that the poverty headcount ratio of Consumable Income is about 10.6%, so without the Targeted Subsidy Program, the value of this indicator would have been about 22.5%. The general results remain unchanged when the Urban-Rural poverty lines are used instead of the $4PPP. The former poverty lines are based on Negahdari, Piraee, Keshavarz Haddad, and Haghighat (2014, 2015) which differentiate between households based on their size and whether they are located in an Urban/Rural area.^18^

**Table 4.** Marginal contribution of taxes and transfers to poverty.

Fiscal Intervention		Marginal contribution to the $4 PPP poverty headcount index of:		Marginal contribution to the Urban-Rural Poverty headcount index of:	
		Disposable Income (0.0939)	Consumable Income (0.1057)	Disposable Income (0.2581)	Consumable Income (0.2805)
Direct Taxes and Contributions	Income Tax	−0.0004	−0.0005	−0.0031	−0.0029
	Employee contributions to the health insurance	−0.0013	−0.0014	−0.0044	−0.0059
	Employer contributions to the health insurance	−0.0008	−0.0005	−0.0045	−0.0053
	Total Direct Taxes and Contributions	−0.0024	−0.0021	−0.0119	−0.0138
Direct Transfers	Targeted Subsidy Program	0.1131	0.1190	0.1473	0.1513
	Social Assistance	0.0104	0.0111	0.0099	0.102
	Semi-cash Transfers (Food)	0.0001	0.0002	0.0002	0.0004
	Total Direct Transfers	0.1217	0.1277	0.1554	0.1591
Indirect Taxes (Sales Taxes)		–	−0.0118	–	−0.0224

Source: Own calculations using the Iranian household survey (1390 Iranian calendar, equivalent to 2011–12).

Note: PPP stands for Purchasing Power Parity. In calculating PPP values, we use the 2005 round of ICP (International Comparison Program) as reported in the World Development Indicators (WDI) published by the World Bank. To change monetary values from the year of survey to 2005, we use the CPI index from the WDI. Urban-Rural poverty lines are based on Negahdari et al. (2014, 2015) which differentiate between households based on their size and whether they are located in an Urban/Rural area.

Now we turn to measuring the effectiveness of taxes and transfers in reducing inequality and poverty. The previous analysis focused on the observed outcome of these fiscal interventions, but what follows provides a context for evaluating the observed marginal contributions. As was mentioned before, these indicators show how effective taxes and transfers are in reducing poverty and inequality when compared to their full potential. Tables 5 and 6 present the results for Impact Effectiveness and Spending Effectiveness, and FI-FGP Effectiveness indices, respectively.

**Table 5.** Impact and spending effectiveness indicators for taxes and transfers in Iran.

Fiscal Intervention		Impact Effectiveness with respect to:			Spending Effectiveness with respect to:		
		Disposable Income	Consumable Income	Final Income	Disposable Income	Consumable Income	Final Income
Direct Taxes and Contributions	Income Tax	0.3445	0.3384	0.3611	0.3461	0.3399	0.3625
	Employee contributions to the health insurance	0.0476	0.0357	0.0765	0.0479	0.0359	0.0770
	Employer contributions to the health insurance	0.1243	0.1152	0.1388	0.1252	0.1161	0.1397
	Total Direct Taxes and Contributions	0.1701	0.1613	0.1901	0.1725	0.1636	0.1925
Direct Transfers	Targeted Subsidy Program	0.3603	0.3648	0.3353	0.2848	0.2872	0.2623
	Social Assistance	0.4122	0.4172	0.4061	0.4022	0.4069	0.3969
	Semi-cash Transfers (Food)	−0.0377	−0.0381	−0.0543	N/A	N/A	N/A
	Total Direct Transfers	0.3747	0.3795	0.3498	0.2943	0.2969	0.2721
Indirect Taxes (Sales Taxes)		*–*	−0.1284	−0.1250	*–*	N/A	N/A
In-kind Transfers	Education Transfers	*–*	*–*	0.2163	*–*	*–*	0.1713
	Education User-fees	*–*	*–*	0.1514	*–*	*–*	0.1530
	Health Transfers	*–*	*–*	0.3002	*–*	*–*	0.2660
	Health User-fees	*–*	*–*	*–*0.2361	*–*	*–*	N/A

Source: Own calculations using the Iranian household survey (1390 Iranian calendar, equivalent to 2011–12).

Note: Fiscal interventions with an N/A are the ones with a negative marginal contribution, so it is mathematically impossible to calculate the spending effectiveness for them.

**Table 6.** FI-FGP effectiveness indicators for taxes and transfers in Iran.

Fiscal Intervention		FI-FGP Effectiveness with respect to ($4PPP):		FI-FGP Effectiveness with respect to (Urban-Rural poverty lines):	
		Disposable Income	Consumable Income	Disposable Income	Consumable Income
Direct Taxes and Contributions	Income Tax	0.9984	0.9964	0.9349	0.9245
	Employee contributions to the health insurance	0.9879	0.9837	0.8719	0.8550
	Employer contributions to the health insurance	0.9964	0.9955	0.9226	0.9075
	Total Direct Taxes and Contributions	0.9945	0.9923	0.9144	0.9009
Direct Transfers	Targeted Subsidy Program	0.1340	0.1492	0.3099	0.3343
	Social Assistance	0.1827	0.2069	0.3589	0.3840
	Semi-cash Transfers (Food)	0.0344	0.0387	0.1293	0.1383
	Total Direct Transfers	0.1464	0.1619	0.3213	0.3456
Indirect Taxes (Sales Taxes)		–	0.9567	–	0.8387
Total System		0.2838	0.4018	0.4174	0.5015

Source: Own calculations using the Iranian household survey (1390 Iranian calendar, equivalent to 2011–12).

Note: PPP stands for Purchasing Power Parity. In calculating PPP values, we use the 2005 round of ICP (International Comparison Program) as reported in the World Development Indicators (WDI) published by the World Bank. To change monetary values from the year of survey to 2005, we use the CPI index from the WDI. The FI-FGT effectiveness indicators are bounded between zero and one and the higher the value of an indicator, the better the tax is in not increasing poverty and a transfer is in reducing poverty. Urban-Rural poverty lines are based on Negahdari et al. (2014, 2015) which differentiate between households based on their size and whether they are located in an Urban/Rural area.

Focusing on Table 5, and with respect to Consumable Income, the Income Tax has the highest Impact Effectiveness of the direct taxes, fulfilling about 34% of its potential in reducing inequality. However, the highest effectiveness belongs to Social Assistance (a direct transfer), which fulfills about 42% of its potential. Among interventions with a positive marginal contribution the lowest Impact Effectiveness belongs to Employee Contributions to the Health Insurance, about 4% of its potential. Health User-fees are the worst: they have an increasing effect on inequality, but compared to Semi-Cash Transfers (Food) and Sales Taxes, which also increase inequality, they have relatively more potential to reduce it.

With regard to Spending Effectiveness, and focusing on the Consumable Income column, Social Assistance (with about 41%) and Income Tax (with about 34%) are the two most effective interventions. Employee Contributions to the Health Insurance are worst, with almost zero effectiveness. That means that with a very small fraction of Employee Contributions to the Health Insurance, one can achieve the same level of reduction in inequality as is currently produced by these contributions. This outcome is expected given the small size of the Marginal Contribution of this intervention (see Table 3).

Table 6 presents FI-FGP effectiveness indicators. As was mentioned earlier, we should not compare Taxes and Transfers because taxes can only increase poverty while transfers can only reduce it. All taxes are highly efficient in raising revenue without significantly increasing poverty, while direct transfers are not very efficient in reducing poverty. Focusing on $4PPP poverty line and among transfers, Social Assistance has the highest effectiveness (about 21% with respect to Consumable Income) and Semi-Cash Transfers have the lowest (about 4% with respect to Consumable Income). The Targeted Subsidy Program’s poverty reduction effectiveness is about 15%. One may question these results for TSP given the high marginal contribution of this program to reducing poverty, as established in the previous sections. But the explanation is in the properties of TSP. The TSP’s cash transfers are made to all Iranians (i.e. poor and non-poor equally), so the total cash transfer is very large, but not specifically targeted toward the poor. As a result, its poverty effectiveness diminishes substantially. Poverty would be reduced significantly if the Targeted Subsidy Program were allocated more toward low-income households. We explore this idea further in the next sub-section. Finally, it is worth noting that the fiscal system as a whole is not very effective in reducing poverty. With respect to Disposable Income and Consumable Income, the fiscal system only realizes about 28% and 39% of its potential, respectively.

The results are generally robust when Urban-Rural poverty lines are used. One should note that these poverty lines are higher than the $4PPP in the urban areas and as a result the effectiveness of the direct transfers, for example, would be automatically higher. However, even with this higher poverty line, direct transfers do not achieve anything more than 35% of their potential to reduce poverty.

### IV.B. Alternative scenarios for implementation of the ‘targeted subsidy program’

Since the TSP makes the largest marginal contribution to the reduction of inequality and poverty, it is important to analyze it further. This cash transfer program (in the survey year used in this paper) offers an identical amount to every Iranian regardless of income (Baseline scenario). In order to be sure our results are not driven by how the income concepts are set up in the Baseline scenario (which uses the income portion of the survey), we reconstruct the same income concepts using the expenditure (of nondurable goods and imputed rent) portion of the survey. We call this Alternative Baseline scenario.

As was mentioned before, the Iranian government has proposed a plan known as the ‘Second Phase’ of the energy subsidies reform (but not yet successfully implemented it) to eliminate eligibility for receiving cash transfer from the top two deciles. What if this new policy had been in place from the beginning? We consider that (i.e. the ‘Second Phase’ policy) as well as two alternative policy scenarios with fiscally neutral effects as compared to the Second Phase policy, asking how much larger the marginal contribution of TSP would be in reducing inequality and poverty.^19^ In the first scenario, we remove the subsidy for the top 40%, but increase transfers to the bottom 60% by about 30% (‘Policy Simulation 1’). In the second scenario, we again eliminate transfers for the top 40%, but increase the cash transfer to those at the bottom 30% by about 60% (‘Policy Simulation 2’). It is important to note that for the two alternative policy simulations, we do not redistribute the cash transfer of the top two deciles so that these two scenarios are fiscally similar to the ‘Second Phase’ scenario.

Panel A in Table 7 shows how the Targeted Subsidy Program’s marginal contribution to reducing inequality changes in different scenarios. The results of the ‘Baseline’ and ‘Alternative Baseline’ cases are very similar indicating that using income or expenditure portions of the household survey to set up the income concepts produces very similar results. As expected as the transfer to the top income groups are removed and the transfer to the low-income group is increased, inequality decreases significantly. Focusing only on Consumable Income, the marginal contribution of TSP to reducing inequality is about 0.0655, 0.0868, and 0.0953 Gini points in the Second Phase and the two alternative scenarios, respectively. To put this in context, note that in the Baseline case the marginal contribution of TSP to the Gini of Consumbale Income is about 0.0552. Therefore, from the inequality perspective, there is not a big difference between the Baseline scenario and the Second Phase, but the two alternative scenarios produce significantly more reduction in inequality.

**Table 7.** Alternative policies for how to manage Targeted Subsidy Program and their effect on inequality and poverty.

**Panel A. Inequality**			
Policy	Marginal contribution to the Gini index of:		
	Disposable Income (DI)	Consumable Income (CI)	Final Income (FI)
Baseline (All income deciles receive the subsidy)	0.0527 (Gini of DI: 0.3686)	0.0552 (Gini of CI: 0.3712)	0.0465 (Gini of FI: 0.3432)
Alternative Baseline: (Baseline with income concepts calculated using reported household expenditure)	0.0647 (Gini of DI: 0.3570)	0.0680 (Gini of CI: 0.3570)	0.0532 (Gini of FI: 0.3140)
Second Phase: No subsidy for top 20%	0.0628 (Gini of DI: 0.3586)	0.0655 (Gini of CI: 0.3609)	0.0559 (Gini of FI: 0.3336)
Policy Simulation 1: No subsidy for top 40% and an extra 30% for bottom 60%	0.0834 (Gini of DI: 0.3379)	0.0868 (Gini of CI: 0.3397)	0.0742 (Gini of FI: 0.3153)
Policy Simulation 2: No subsidy for top 40% and an extra 60% for bottom 30%	0.0916 (Gini of DI: 0.3297)	0.0953 (Gini of CI: 0.3312)	0.0816 (Gini of FI: 0.3080)

**Panel B. Poverty**				
Policy	Marginal contribution to the $4 PPP poverty headcount index (PHI) of:		Marginal contribution to the Urban-Rural poverty headcount index (PHI) of:	
	DI	CI	DI	CI
Baseline (All income deciles receive the subsidy)	0.1131 (PHI of DI: 0.0939)	0.1190 (PHI of CI: 0.1057)	0.1473 (PHI of DI: 0.2581)	0.1513 (PHI of CI: 0.2805)
Alternative Baseline (Baseline with income concepts calculated using reported household expenditure)	0.1501 (PHI of DI: 0.1211)	0.1602 (PHI of CI: 0.1348)	0.1578 (PHI of DI: 0.3662)	0.1586 (PHI of CI: 0.3923)
Second Phase: No subsidy for top 20%	0.1131 (PHI of DI: 0.0939)	0.1190 (PHI of CI: 0.1057)	0.1473 (PHI of DI: 0.2581)	0.1512 (PHI of CI: 0.2806)
Policy Simulation 1: No subsidy for top 40% and an extra 30% for bottom 60%	0.1387 (PHI of DI: 0.0682)	0.1469 (PHI of CI: 0.0778)	0.1832 (PHI of DI: 0.2222)	0.1908 (PHI of CI: 0.2410)
Policy Simulation 2: No subsidy for top 40% and an extra 60% for bottom 30%	0.1578 (PHI of DI: 0.0492)	0.1679 (PHI of CI: 0.0568)	0.1819 (PHI of DI: 0.2236)	0.1837 (PHI of CI: 0.2481)

Source: Own calculations using the Iranian household survey (1390 Iranian calendar, equivalent to 2011–12).

Note: PPP stands for Purchasing Power Parity. In calculating PPP values, we use the 2005 round of ICP (International Comparison Program) as reported in the World Development Indicators (WDI) published by the World Bank. To change monetary values from the year of survey to 2005, we use the CPI index from the WDI. Urban-Rural poverty lines are based on Negahdari et al. (2014, 2015) which differentiate between households based on their size and whether they are located in an Urban/Rural area.

Panel B in Table 7 performs a similar analysis under each scenario using the poverty headcount ratio for the change in poverty. The Baseline and the Second Phase are not different, given that the top 20% would not become poor if they lose this cash transfer. The Baseline and Alternative Baseline scenarios have very similar values, especially when the Urban-Rural poverty lines are used. For simulated scenarios and with respect to Consumable Income and $4PPP poverty line, Policy Simulation 1 and 2 improve the marginal contribution of this cash transfer from 11.90 percentage points in the Baseline to 14.69 percentage points and 16.79 percentage points respectively. The poverty headcount ratio decreases from about 11% in the Second Phase case to about 6% in Policy Simulation 2; that is a significant reduction in poverty for a fiscally neutral policy alternative. The change in poverty line from $4PPP to the Urban-Rural poverty lines do not change our conclusions and the results are very similar as is clear in Panel B of Table 7.

The poverty-reducing effect of an additional cash transfer to low income deciles is significant. To get at that effect, we analyze how different policy scenarios change the poverty headcount index of urban versus rural areas. These results are presented in Appendix C. Overall, TSP substantially benefits the rural areas.

Table 8 presents the effectiveness of TSP under different scenarios, taking the values reported for the Baseline scenario from the previous tables for comparison purposes. The Baseline and Alternative Baseline produce a very similar set of results. With regard to all measures of effectiveness, eliminating the cash transfer from the top deciles and allocating it to the low-income groups improves the performance of the TSP significantly. In fact, Policy Simulation 2, which has the most focused approach to allocating the cash transfer to low-income households, almost doubles the effectiveness of the Baseline scenario in reducing inequality. Still, the FI-FGP effectiveness indicator reveals that even this scenario has significant room for improvement, because it only reaches about 22% of its potential when the $4PPP poverty line is used. Changing the poverty line to the Urban-Rural poverty lines increases the effectiveness of TSP in all scenarios but this is a byproduct of the fact that the Urban-Rural poverty lines are higher than the $4PPP line in the urban areas.

**Table 8.** Effectiveness of targeted subsidy program in alternative policy scenarios.

**Panel A. Impact Effectiveness**			
Policy	Impact Effectiveness with respect to:		
	Disposable Income	Consumable Income	Final Income
Baseline	0.3603	0.3648	0.3353
Alternative Baseline	0.3804	0.3852	0.3468
Second Phase	0.4850	0.4891	0.4586
Policy Simulation 1	0.6447	0.6479	0.6103
Policy Simulation 2	0.7077	0.7108	0.6709

**Panel B. Spending Effectiveness**			
Policy	Spending Effectiveness with respect to:		
	Disposable Income	Consumable Income	Final Income
Baseline	0.2848	0.2872	0.2623
Alternative Baseline	0.2874	0.2890	0.2572
Second Phase	0.4111	0.4133	0.3852
Policy Simulation 1	0.5747	0.5764	0.5377
Policy Simulation 2	0.6435	0.6452	0.6025

**Panel C. FI-FGP Effectiveness**				
Policy	FI-FGP Effectiveness with respect to ($4PPP):		FI-FGP Effectiveness with respect to (Urban-Rural poverty lines):	
	Disposable Income	Consumable Income	Disposable Income	Consumable Income
Baseline	0.1340	0.1492	0.3099	0.3343
Alternative Baseline	0.2050	0.1138	0.4574	0.3444
Second Phase	0.1586	0.1766	0.3669	0.3957
Policy Simulation 1	0.1798	0.2012	0.4393	0.4747
Policy Simulation 2	0.1921	0.2160	0.4769	0.5103

Source: Own calculations using the Iranian household survey (1390 Iranian calendar, equivalent to 2011–12).

Note: The description of policy scenarios are as follows. Baseline: all income deciles receive the subsidy; Alternative Baseline: The same as Baseline with income concepts calculated using reported household expenditures; Second Phase: No subsidy for top 20%; Policy Simulation 1: No subsidy for top 40% and an extra 30% for bottom 60%; Policy Simulation 2: No subsidy for top 40% and an extra 60% for bottom 30%. PPP stands for Purchasing Power Parity. In calculating PPP values, we use the 2005 round of ICP (International Comparison Program) as reported in the World Development Indicators (WDI) published by the World Bank. To change monetary values from the year of survey to 2005, we use the CPI index from the WDI. Urban-Rural poverty lines are based on Negahdari et al. (2014, 2015) which differentiate between households based on their size and whether they are located in an Urban/Rural area.

## V. Conclusion

This paper analyzes the effect of different components of the fiscal system in Iran on reducing inequality and poverty. Using the marginal contribution approach, we show that direct transfers in general, and the (cash component of the) Targeted Subsidy Program in particular, play the most significant role in creating a more equal distribution of income and reducing poverty in Iran. The system as a whole reduces the inequality of income distribution by about 20% (comparing Market Income to Final Income) and the poverty head count ratio by about 50% (comparing Market Income to Consumable Income). The Targeted Subsidy Program alone reduces the inequality and poverty of Consumable Income by about 0.0552 Gini points and 12 percentage points respectively (using $4PPP as the poverty line). The main reduction in poverty comes from the rural areas: this program reduces the poverty headcount ratio from about 44% to 23%. The urban areas only experience a moderate 8 percentage point reduction in poverty (i.e. from 13% to 5%) due to this program.

We find mixed results for how effective taxes and transfers are in reducing inequality and poverty compared to their potential. Taxes are very effective in raising revenue without increasing poverty and are moderately effective in reducing inequality. On the other hand, transfers exhibit a similar, moderate effectiveness in reducing inequality to that of taxes, but they are not focused on poor households, and realize less than 17% of their potential power to reduce poverty.

We evaluate different policy scenarios about how to proceed with the current Targeted Subsidy Program in Iran. We find that if the Iranian government’s current plan to eliminate the cash transfer of top deciles were extended from the top 20% to the top 40%, and were combined with a moderate increase in the cash transfer to the bottom deciles, the additional reduction in poverty and inequality would be considerable. If the cash transfer of the top 40% is eliminated and the cash transfer to the bottom 60% is increased by only 30%, inequality and poverty would be reduced by an additional 8.5% and 26.4%, respectively (compared to the current Gini and poverty headcount ratio of Consumable Income). This poverty reduction effect would not be the same for rural versus urban areas. An extra 30% going to the bottom 60% of the income distribution would reduce the poverty headcount ratio of Consumable Income to 16.7% (from 22.8%) in rural areas. In urban areas, the reduction in the poverty head count ratio would be only 1.4 percentage points (i.e. 4.2% from 5.6% now). The power of the Targeted Subsidy Program in reducing inequality and poverty stems from the ability of the program to reach the bottom deciles of the income distribution in rural areas of Iran. Therefore, the main policy recommendation of this paper is to not just remove the cash transfers from the top 20% (as it was implemented recently in Iran), but extend it to the top 40% and to allocate part of the resulting extra funds to the bottom deciles, especially in the rural areas.

## Supplementary Material

Supplemental Material

## Notes

1 Energy here refers to subsidies on electricity, water, natural gas, and oil-based fuels.

2 The government justified this reform on two main grounds: the high fiscal burden of the energy subsidies, which amounted to 20% of GDP in 2010 (or $70 billion US dollars), and the fact that fiscal resources disproportionately were benefitting the non-poor given that the latter consume much more of the subsidized goods (Guillaume et al., 2011; Salehi-Isfahani et al., 2015; Mostafavi-Dehzooei & Salehi-Isfahani, 2017). In addition to the fiscal burden and the failure of the energy subsidies to target the poor, other justifications also have been used to gain public support for this reform: the excessive amount of energy consumption per GDP as compared to Iran’s neighbors and other developing countries, the excessive waste in the use of subsidized goods, the environmentally negative side effects of cheap fossil fuels, the problem of smuggling subsidized fuel out of the country, and the fear of a potential international embargo on importing gasoline (a main fuel for cars) precipitating a need to reduce consumption of this product (Guillaume et al., 2011; Salehi-Isfahani et al., 2015).

3 For a more general equilibrium analysis of the effect of removing subsidies see Gahvari and Taheripour (2011).

4 Atamanov, Mostafavi, Salehi Isfahani, and Vishwanath (2016) and Salehi-Isfahani (2017) also find a decrease in inequality and poverty in the first couple of years following the reform. However, their analysis describes general trends of these indicators and not exclusively the role of the subsidy reform.

5 The estimated total cash transfer for this period (December 2010–June 2012) is about 62,000 billion Rials (about $5.4 billion) and the government revenue from the increase in prices is about 30,000 billion Rials (about $2.7 Billion). The dollar values in parentheses are based on the average exchange rate for this period from the Central Bank of Iran (CBI, 2014).

6 Note that in both policy simulations, the cash transfer of the top two deciles is completely removed from the fiscal system and not redistributed to the other deciles.

7 The tax incidence literature includes a long list of studies with empirical estimates going back more than half a century (Musgrave, 1959; Musgrave, Carroll, Cook, & Frane, 1951; Musgrave, Case, & Leonard, 1974; Pechman & Okner, 1974). Similarly, on the expenditure side, there are decades of work using the traditional approach (Meerman, 1979; Selowsky, 1979) and a behavioral approach (Gertler & Glewwe, 1990; Gertler & van der Gaag, 1990; Younger, Sahn, Haggblade, & Dorosh, 1999). For more recent work see, for example: Alm and Wallace (2007), Martinez-Vazquez (2008), Förster and Whiteford (2009), Immervoll and Richardson (2011), Bucheli, Lustig, Rossi, and Amábile (2014), Higgins and Pereira (2014), Jaramillo (2014), Lustig and Pessino (2014), Arauco, Molina, Pozo, and Aguilar (2014), Scott (2014), Cabrera, Lustig, and Morán (2015), Higgins and Lustig (2016), Higgins, Lustig, Ruble, and Smeeding (2016), Lustig (2015, 2016a, 2016b), Younger, Myamba, and Mdadila (2016), and Younger, Osei-Assibey, and Oppong (2017).

8 For example, the marginal contribution of direct taxes to reducing inequality is measured by comparing the Gini of the system with direct taxes to the Gini of the same system without direct taxes. One also can think of this counter-factual as having the tax or transfer replaced with an alternative tax or transfer of the same size but with no effect on inequality or poverty.

9 Lambert (2001) and Enami et al. (2018) show this mathematically. Also, Enami (2018a) shows what happens when taxes and transfers end up reranking individuals.

10 Unless otherwise specified, throughout this paper we use $4 per day in 2005 purchasing power parity (PPP) as the poverty line.

11 We calculate the poverty indices using the international poverty lines defined without accounting for the ‘consumption’ of education and health. To be consistent with the definition of these poverty lines, we do not include the in-kind transfers for education and health as part of the fiscal system when evaluating its effect on poverty.

12 Note that these estimates rely on the concept of Consumable Income which is described later in Section III.

13 The survey actually includes pre-tax income for employees. For the self-employed, market income is generated by subtracting Business Costs from Sales since both items are in the survey.

14 Or replacing that tax (or transfer) with another tax (or transfer) that is neutral in reducing inequality (or poverty).

15 Fellman et al. (1999) call a tax (transfer) optimal when it optimizes the social welfare index of interest (e.g. Gini index or poverty head count ratio) comparing to the class of all taxes (transfers) that raise (distribute) an identical amount of funds.

16 Most of the survey data is available at goo.gl/MnYB23. Please note that the online database does not include the survey weight variables. These variables are, however, available for researchers who visit the Statistical Center of Iran in person.

17 Results related to education and health in-kind transfers should be interpreted with caution as the quality of service that a household receives is not incorporated in imputation of these two transfers (please see Appendix A for more detail). However, this has no impact on the fact that given the current values used in this analysis, we observe a Lambert Conundrum in our database.

18 We use the equivalent US 2005 $PPP of poverty lines in Negahdari et al. (2014, 2015) in our analysis. Depending on the size of a household, the poverty line for an urban household ranges from $7.14 PPP to $8.93 PPP. For the rural households, the range of values is from $3.19 PPP to $3.51 PPP. The exact poverty line used for each size of household in urban and rural areas is available upon request.

19 These microsimulations follow the ‘arithmetical approach’ that ignores the behavioral responses. For more information about this type of simulation see Bourguignon and Spadaro (2006).
