# Supplementary material for: Fiscal policy, inequality, and poverty in Iran: assessing the impact and effectiveness of taxes and transfers
Source: Middle East Dev J. 2019 Mar 8;11(1):49–74. doi: 10.1080/17938120.2019.1583510 (PMC6743812; doi:10.1080/17938120.2019.1583510)
Supplement: Supplemental Material [file RMDJ_A_1583510_SM4710.docx]

**Appendix A: Description of Iran’s Fiscal system.**

This appendix includes three subsections describing taxes, transfers, and pension system in Iran.

**A.1. Taxes**

In Iran, the current tax system has two main categories: direct and indirect taxes. The two main sub-categories of direct taxes are property^[[1]](#footnote-1)^ and income^[[2]](#footnote-2)^ taxes. The main indirect tax in Iran is the Value-added tax (VAT) (INTA, 2015).^[[3]](#footnote-3)^ The movement from sales tax to VAT is a recent policy reform in Iran; it was not implemented fully in the year of the survey that is used in this study (i.e. 2011-2012). It is worth noting that in Iran the main entity in charge of taxation is the Ministry of Finance and Economic Affairs.

In this paper, we mainly focus on estimating the incidence of taxes that can be directly observed in the household survey, or inferred or simulated from the available data. For example, the income tax of self-employed individuals is directly observed in the survey. However, payroll taxes are calculated using the reported gross and net income variables, as well as the reported deductions for pensions and health insurance schemes. We simulate the incidence of sales taxes using the general rule of 3% sales tax^[[4]](#footnote-4)^ combined with data on household monthly consumption expenditures to impute the value of sales taxes for the whole year.^[[5]](#footnote-5)^ We exclude corporate income taxes and stamp duties from our analysis.

**A.2. Transfers**

Iran has several transfer programs and subsidies. we classify them by three main categories: cash or near-cash transfers, in-kind transfers, and price subsidies. The latter group, which includes consumption and production subsidies, is not included in this analysis because we cannot identify the beneficiary households in the survey.

*Cash or near-cash transfers*

The first category, “cash transfer” programs, includes the Targeted Subsidy Program,^[[6]](#footnote-6)^ cash transfer programs by BSOI^[[7]](#footnote-7)^ (which is an organization in charge of providing assistance to the families of those who are considered “martyrs, prisoners of war or injured in defending the Islamic revolution in Iran”), the Imam Khomeini Relief Foundation^[[8]](#footnote-8)^ (which mainly assists low income families), the Islamic Revolution Mostazafan Foundation^[[9]](#footnote-9)^ (which mainly assists low income families), and the State Welfare Organization of Iran^[[10]](#footnote-10)^ (which assists several groups, including individuals who are disabled, addicted, orphans, or elderly).

Cash transfers received through the TSP are directly observed in the survey. It shows that almost all of the households (about 96%) receive this subsidy. In the survey year, beneficiaries received 455,000 Rials per person per month (equivalent to $37 to $44 depending on the exchange rate from March 2012 or March 2011, respectively). Moreover, the average transfer received by an Iranian household^[[11]](#footnote-11)^ through the TSP is about 14.7 million Rials (about 15% of average “Market Income^[[12]](#footnote-12)^” of a household). To implement this subsidy reform, a new organization called the Targeting Subsidies Organization was established. The transfer is deposited into the bank account of the head of the household, and ATM machines were installed in remote rural areas to facilitate access to this transfer.

All of the other cash transfer programs mentioned above are reported in the survey as a total amount, without distinguishing among them. We call these combined transfers the “Social Assistance” program. The average transfer received by an Iranian household through the Social Assistance program is 0.9 million Rials (about 0.9% of the average Market Income of a household). The third type of transfer programs included in the analysis are food, or so-called near-cash transfers: the edible goods that a household receives for free, but not from other households. The expenditure data has a code to identify goods that are consumed “free but not from other households.” Given the existence of this “self-consumption” code, we decide to consider these free edible goods as being provided by the government. The average transfer received by an Iranian household as free food is about 0.06 million Rials (about 0.07% of the average Market Income of a household).

*In-kind transfers*

In-kind transfers are divided into education, health, and housing transfers. The latter category is not included in this analysis because we cannot identify the beneficiary households in the survey.

Primary and secondary education in Iran are under the supervision of the Ministry of Education. There are 12 grades: 5 for primary, 3 for middle school, and 4 for high school. Compulsory education runs until the end of middle school (i.e. eighth grade). Primary and secondary education are free for all 12 grades in public schools, but people have the option to switch to private schools. Tertiary education is supervised by the Ministry of Science, Research and Technology and by the Ministry of Health and Medical Education, depending on the field of study. Tertiary education is not free, but public universities offer it freely in exchange for an obligation that a student will work in the country for some period after the end of their education. This could be as long as three times the length of their education.^[[13]](#footnote-13)^ However, students, have the option of paying for their degrees from the Ministry of Science, Research and Technology before their obligation ends and leaving the country; this is harder for those who fall under the jurisdiction of the Ministry of Health and Medical Education. In addition to these education-for-work type of universities, there are both public and private universities that admit students who are willing to pay for their education. Our analysis includes education transfers calculated by using the imputation method and the per-pupil budgetary expenditures on education (as reported in Adlband, 2011; MNA, 2011).^[[14]](#footnote-14)^ Since the household survey does not include the institutional households, the tertiary subsidies allocated through our method potentially underestimates the extend to which rich households receive it. Moreover, since the education subsidies are imputed identically for all households with students attending a specific grade regardless of the actual quality of education that they receive in their area, the interpretation of the results should be done with cautious.

Finally, in terms of its health care system, Iran combines medical care and education through both public and private medical schools. Each province of Iran has at least one public medical university, which is a place to train physicians and is responsible for public health in that province. These universities, which are directly supervised by the Ministry of Health and Medical Education, control a health network that expands into the rural and urban areas of each province. Every village, or a group of them, has a “health house” (with the ratio of one health house per 1,200 residents) with a trained health worker known as a “Behvarz.” The health houses are all connected to “rural health centers” (with the ratio of one rural health center to 7,000 residents), each with at least one physician. A similar structure exists in the urban areas, where “health posts” and “urban health centers” respectively replace the corresponding entities in the rural areas. All of the rural and urban health centers are supervised by “district health centers” which are controlled by the public medical university in charge of the province. Public hospitals also directly report to this university. In addition to the public health system, the private sector is active in the field, with private physician offices and hospitals. Moreover, NGOs are also present and active in Iran’s health care market (Asaei, 2015; Mehrdad, 2009).

Medical services are not free in Iran, but they receive a subsidy from the government. The government’s budget has a specific line for a “medicine and skim milk” subsidy which amounted to 3,900 billion Rials in 1390 (2011-12), the year of the survey used in this analysis. Health insurance is available to a large fraction of population, but mostly involves large copayments. According to the Statistical Center of Iran, the total public expenditure on health in year 1390 (2011-12) was about 170,000 billion Rials (SCI, 2015). In that year, the private expenditure was about 283,000 billion Rials; the households’ share was about 245,000 billion Rials. The balance was covered by private insurance, employers, NGOs, and the additional (optional) coverage provided by the public insurance companies. Finally, international sources contributed 26,000 billion Rials to health expenditures in Iran in that year (SCI, 2015). For the purpose of this analysis, we allocate the per capita health transfer of 2,250,720 Rials (which is the per capita health expenditure in the year of survey) to every member of a household that has a medical expenditure in the survey.^[[15]](#footnote-15)^ Similar to education subsidies results of the impact of health subsidies on inequality should be interpreted with caution since the quality of care that a household has access to is not included in the imputation of the subsidy that it receives.

**A.3. Pension system**

The first civil servant (contributory) pension system legislation in Iran dates back to 1922 (1301 on the Iranian calendar) (CSPO, 2015). Since then, it has experienced several major changes, but it is still mainly a pay-as-you-go (PAYG) system and is known as the Civil Servants Pension Organization (CSPO). Currently, there are several ways through which a civil servant can be retired. These include “compulsory retirement” (for employees 65 years of age regardless of the years of rendering service), “retirement based on mutual agreement” (for employees who are 50 years of age and have rendered service for at least 25 years if male and 20 years if female), “forcible retirement” (which is based on the verdicts issued by the board of investigation of administrative violations, but requiring 25 years of service for males and 20 years for females), “voluntary retirement by authority of employee” (if of age or by authority of the organization if the employee has rendered 30 years of service), and “invalidity pension” (for those experiencing a non-occupation related invalidity). The main factors in calculating one’s pension are years of service and salary and benefits in the final two years of service (CSPO, 2015).

Military members have their own pension and health insurance system. Prior to 2002 (1381 on the Iranian calendar), the different branches of Iran’s armed forces had their own pension systems, but they were combined in to one organization in that year (although the funds of each branch are still kept separate from each other). This “Retirement Organization of Armed Forces” is part of the “Social Security Organization of Armed Forces,” the centralized entity in charge of armed forces welfare. This system is PAYG and is mainly funded through fees paid by the military members and the government, a governmental budget, and the financial investments of the Organization (IPRS, 2015).

Those who are employed in the private sector are mandated to be covered by the pension and health insurance system provided by the Social Security Organization (SSO). Social security was first provided to workers in 1932 (1310 on the Iranian calendar) (SSO, 2015). This system is also PAYG, and is considered an independent organization under the supervision of the “Ministry of Cooperatives, Labour and Social Welfare.” SSO is financed through payments made by employees (7% of their base salary), employers (about 23% of the base salary of each employee), and government (3% of the base salary of each insured employee), as well as financial activities by the entities that are controlled by SSO (SSO, 2015). Any employees who are covered by the SSO are considered “insured” employees, and the fees that are paid by them and their employer are also called an “insurance fee.” This is mainly because SSO provides both health and retirement insurance (i.e. pensions), as well as other types of insurance (e.g. invalidity and unemployment) (SSO, 2015).^[[16]](#footnote-16)^ Those who are self-employed have the option of self-insuring through SSO. The general rule for the calculation of a pension in SSO is similar (although not identical) to that of CSPO. Male and female employees have to be at least age 50 and 45, respectively, and must have at least 30 years of paid insurance fees to be eligible for retirement. The age requirement does not apply to those who have at least 35 years of paid insurance fees. Men and women who are over age 60 and 55, respectively, and who have at least 20 years of paid insurance fees, are eligible to become retired. Under some special circumstances, women can be eligible for retirement if they are at least 42 years old (SSO, 2015).

It is important to note that all incomes from pensions are exempt from taxes (CSPO, 2015). Moreover, the pension deduction for all Civil and Military servants is 9% of their salary, and the government pays 1.5 times their fee as its contribution to the pension funds (HVM, 2015). The household survey that we use in this study has information about the pension that is received by any member of a household, as well as the deductions for the social security system and the related health insurance.

**Appendix B: Methodological Appendix**

The first step in fiscal incidence analysis is to construct the basic income concepts. Figure B1 is the same as Figure 1 in the main text and presents the income concepts used in this paper. Table B1 describes the components included in these income concepts in the case of Iran. We begin with Market Income.^[[17]](#footnote-17)^ We then subtract direct taxes and add cash transfers to obtain Disposable Income. Next, we subtract indirect taxes to generate Consumable Income. (Because consumption subsidies were replaced by the TSP, there are no consumption subsidies). Finally, we add the monetized value at average government cost of In-kind Transfers (i.e. health and education), net of user fees, to obtain Final Income. Table B2 describes the main assumptions used to allocate taxes and transfers at the household level, and is based on the standardized table developed by the Commitment to Equity Institute at Tulane University. This standardization makes the current paper comparable to 23 completed and 14 in-progress incidence analysis projects; their results are part of the CEQ Institute Data Center on Fiscal Redistribution.

**Figure B1: A framework to define income concepts and combine fiscal interventions.**

**Market Income**

(Factor Income plus Pensions minus Contributions to Pensions)

**+**

Direct transfers

**-**

Direct taxes

**Disposable Income**

Indirect taxes

**-**

**Consumable Income**

Monetized value of education and health services (in-kind transfers)

**Final Income**

**-**

**+**

Co-payments and user fees for education and health services

**Net Market Income**

**Gross Income**

Direct taxes

**-**

**+**

Direct transfers

Source: Lustig (2018) with some adaptation.

Note: Core Income Concepts in dark blue background, Fiscal Interventions in white background.

**Table B1. Description of Market Income and other income components**

| **Main Categories** | **Sub Categories** | **Description** |
| --- | --- | --- |
| **Market Income** | Factor Income | All monetary and non-monetary income received as an employee or self-employed individual excluding any subsidy or social assistance and including imputed rent for home owners. All components are directly observed in the survey. |
|  | Contributory Pensions | All pensions received through the retirement programs. The relevant information is observed directly in the survey. |
|  | Employee contributions to the Social Security Insurance | The deductions from employees’ paychecks that is paid for the social security insurance (i.e. pension) of an employee. The relevant information is observed directly in the survey. |
|  | Employer contributions to the Social Security Insurance | The employers’ payment toward the social security insurance (i.e. pension) of employees. Since this is a mandatory payment and we assume it results in lower payments to employees, we include it as a type of deduction. The relevant information is observed directly in the survey. |
| **Direct Taxes and Contributions** | Income Tax | Income tax for self-employed individuals (observed directly in the survey) and payroll tax for employees (imputed using the data about gross and net income as well as contributions to pensions). |
|  | Employee contributions to the health insurance | The deductions from employees’ paychecks that is paid toward the health insurance. The relevant information is observed directly in the survey. |
|  | Employer contributions to the health insurance | The employers’ payment toward the health insurance of employees. Since this is a mandatory payment and we assume it results in lower payments to employees, we include it as a type of deduction. The relevant information is observed directly in the survey. |
| **Direct Transfers** | Targeted Subsidy Program | The direct cash transfer program that is established by the government following the energy subsidy reform in Iran. The relevant information is observed directly in the survey. |
|  | Social Assistance | Includes all cash transfers to low income individuals through public organizations. The relevant information is observed directly in the survey. |
|  | Semi-cash Transfers (Food) | Include the monetary value of all edible items that a household receives for free. The values are imputed assuming all edible goods that are obtained “free but not from other households” are provided by the different public agencies. |
| **Indirect Taxes** | - | Sales taxes. Imputed using the 3% statutory rate (which is applicable to most of goods) and the information available in the survey about the consumption expenditure of each household) |
| **In-kind Transfers** | Education | Includes a nominal subsidy for each student in a household depending on the grade minus any user fees (the latter is observed directly in the survey) |
|  | Health | Includes a nominal subsidy for each individual in a household with health costs minus these costs (the latter is observed directly in the survey) |

**Table B2. Key assumptions for fiscal incidence analysis in Iran.**

| **Assumption type** | **Iran (2011-12)** |
| --- | --- |
| *Use of input-output matrix for indirect taxes and subsidies* | |
| Indirect taxes calculated with an input-output matrix? | No |
| Indirect subsidies calculated with an input-output matrix? | No |
| *Take-up assumptions* | |
| Take-up of direct transfers—as reported in the survey? | Yes |
| Take-up of in-kind transfers—as reported by use? | Yes |
| *Social security contributions, taxes, and subsidies (shifting assumptions)* | |
| Social security contributions: only those paid by employee or both those paid by employee and employer are assumed to be borne by employee? | Both borne by employees |
| Direct taxes: burden of direct personal income taxes assumed to be borne entirely by the recipient of income? | Yes |
| Indirect taxes: burden assumed to be borne entirely by the consumer? | Yes |
| Indirect subsidies: benefit assumed to be received entirely by the consumer? | n.a. |
| *Tax evasion assumptions* | |
| Direct taxes | Informal employees assumed to pay no personal income tax |
| Indirect taxes | Zero evasion is assumed: The most common sales tax rate (3%) is applied to all taxable goods consumed by a household |
| *Scaling-up or scaling-down assumptions* | |
| In-kind education and health services scaled up or down? | Scaled down |
| Other taxes, transfers, and subsidies individually (such as taxes on tobacco) or as a category (such as total indirect taxes) scaled up or down? | Scaled down |
| *Treatment of administrative costs and capital expenditures* | |
| Cash and near-cash transfers include administrative costs? | No |
| Education and health include administrative costs? | No |
| Education and health include capital expenditures? | No |

*Note:* n.a. = not applicable.

**Appendix C: The Poverty Reduction Impact of Targeted Subsidy Program Alternative Policies: Urban vs. Rural**

Panels A and B of Table C1 present the effect of alternative policies in treating Targeted Subsidy Program (TSP) on urban and rural areas respectively. These two panels demonstrate that TSP substantially benefits rural areas. In the baseline case and the second phase, the TSP reduces the poverty headcount ratio (for the Consumable Income) in rural areas by about 22 percentage points as opposed to only 8 percentage points in urban areas. Removing the cash transfers from the top deciles and allocating it to the bottom deciles (in scenarios one and two) causes the marginal contribution of TSP to reducing poverty in rural areas to increase. In the rural areas, the marginal contribution of the TSP to the poverty headcount index increases from about 21.88 percentage points in the baseline case to about 28.03 and 32.96 percentage points in scenarios one and two respectively. In the urban areas, the TSP’s marginal contribution increases from about 7.82 percentage points to only 9.23 and 10.17 percentage points in these two scenarios.

**Table C1. Alternative policies for how to manage Targeted Subsidy Program and their effect on poverty in urban vs. rural areas**

**Panel A. Urban areas**

| **Policy** | **FI-FGP Effectiveness with respect to ($4PPP):** | | **FI-FGP Effectiveness with respect to (Urban-Rural poverty lines):** | |
| --- | --- | --- | --- | --- |
|  | **Disposable Income** | **Consumable Income** | **Disposable Income** | **Consumable Income** |
| Baseline (All income deciles receive the subsidy) | 0.0720  (PHI of DI: 0.0482) | 0.0782  (PHI of CI: 0.0556) | 0.1197  (PHI of DI: 0.3132) | 0.1220  (PHI of CI: 0.3366) |
| Alternative Baseline (Baseline with income concepts calculated using reported household expenditure) | 0.1154  (PHI of DI: 0.0671) | 0.1274  (PHI of CI: 0.0772) | 0.1249  (PHI of DI: 0.4489) | 0.1212  (PHI of CI: 0.4778) |
| Second Phase: No subsidy for top 20% | 0.0720  (PHI of DI: 0.0482) | 0.0782  (PHI of CI: 0.0556) | 0.1197  (PHI of DI: 0.3132) | 0.1220  (PHI of CI: 0.3366) |
| Policy Simulation 1: No subsidy for top 40% and an extra 30% for bottom 60% | 0.0846  (PHI of DI: 0.0356) | 0.0923  (PHI of CI: 0.0415) | 0.1527  (PHI of DI: 0.2802) | 0.1575  (PHI of CI: 0.3011) |
| Policy Simulation 2: No subsidy for top 40% and an extra 60% for bottom 30% | 0.0926  (PHI of DI: 0.0276) | 0.1017  (PHI of CI: 0.0320) | 0.1404  (PHI of DI: 0.2925) | 0.1354  (PHI of CI: 0.3232) |

**Panel B. Rural areas**

| **Policy** | **FI-FGP Effectiveness with respect to ($4PPP):** | | **FI-FGP Effectiveness with respect to (Urban-Rural poverty lines):** | |
| --- | --- | --- | --- | --- |
|  | **Disposable Income** | **Consumable Income** | **Disposable Income** | **Consumable Income** |
| Baseline (All income deciles receive the subsidy) | 0.2134  (PHI of DI: 0.2055) | 0.2188  (PHI of CI: 0.2280) | 0.2146  (PHI of DI: 0.1238) | 0.2227  (PHI of CI: 0.1437) |
| Alternative Baseline (Baseline with income concepts calculated using reported household expenditure) | 0.2350  (PHI of DI: 0.2527) | 0.2401  (PHI of CI: 0.2754) | 0.2383  (PHI of DI: 0.1644) | 0.2498  (PHI of CI: 0.1834) |
| Second Phase: No subsidy for top 20% | 0.2134  (PHI of DI: 0.2055) | 0.2188  (PHI of CI: 0.2280) | 0.2146  (PHI of DI: 0.1238) | 0.2227  (PHI of CI: 0.1437) |
| Policy Simulation 1: No subsidy for top 40% and an extra 30% for bottom 60% | 0.2710  (PHI of DI: 0.1479) | 0.2803  (PHI of CI: 0.1665) | 0.2577  (PHI of DI: 0.0807) | 0.2720  (PHI of CI: 0.0944) |
| Policy Simulation 2: No subsidy for top 40% and an extra 60% for bottom 30% | 0.3170  (PHI of DI: 0.1019) | 0.3296  (PHI of CI: 0.1172) | 0.2830  (PHI of DI: 0.0553) | 0.3015  (PHI of CI: 0.0649) |

**Source:** Own calculations using the Iranian household survey (1390 Iranian calendar, equivalent to 2011-12).

**Note:** PPP stands for Purchasing Power Parity. In calculating PPP values, we use the 2005 round of ICP (International Comparison Program) as reported in the World Development Indicators (WDI) published by the World Bank. To change monetary values from the year of survey to 2005, we use the CPI index from the WDI. Urban-Rural poverty lines are based on Negahdari et al. (2014, 2015) which differentiate between households based on their size and whether they are located in an Urban/Rural area.

**References**

Adlband, Tahereh. 2011. “1390 Budget Bill from the Education Perspective” (tentative translation from Farsi title: “Layehe-ye Boodje-ye Saal-e 1390-e Kol-e KeshvarazNegah-e AmoozeshvaParvaresh”). Barname weekly, Year 9, Number 405: 1-9.

Asaei S. E. 2015. “Iran’s Excellent Primary Health Care System”. UNICEF. Information extracted from the following links on August 05, 2015: <http://www.unicef.org/iran/media_4427.html>

Asre Bank News Agency. 2014. “The comparative table of price change in the 1^st^ and 2^nd^ phase of the Targeted Subsidy Reform” (a rough translation from the original title in Farsi). April 26, 2014: <http://goo.gl/3gfe1U>

CSPO (The Civil Servants Pension Organization, Iran). Information extracted from the following links on August 05, 2015. <http://www.retirement.ir/English/History/RetirementHistory.aspx> , <http://www.retirement.ir/English/Law/ObtainingRetirementPension.aspx> , <http://www.retirement.ir/English/Law/AmountRetirementPension.aspx> , <http://www.retirement.ir/EvaluatePension.aspx>

HVM (Hoghoghi-Va-Majles: Legal and Parliamentary Affair Office of the Judicial Branch, Iran) Information extracted on August 05, 2015. <http://hvm.ir/lawprint.asp?id=31013>

INTA (Iranian National Tax Administration). Information extracted on August 05, 2015. <http://en.intamedia.ir/>

IPRS (Islamic Parliament Research Center, Iran). Information extracted on August 05, 2015. <http://rc.majlis.ir/fa/law/show/135743>

Iranian Labour News Agency (ILNA). 2013. The related report can be found in the following link: <http://goo.gl/rz3a6V>

Lustig, Nora (Ed.). 2018. ‘Commitment to Equity Handbook: Estimating the Impact of Fiscal Policy on Inequality and Poverty’. Brookings Institution Press and CEQ Institute, Tulane University.

Mehrdad, Ramin. 2009. “Health system in Iran.” JMAJ 52, no.1: 69-73. Available at: <http://www.med.or.jp/english/pdf/2009_01/069_073.pdf>

MNA (Mehr News Agency, Iran). 2011. The related report can be found in the following link: <http://goo.gl/YM3v2I>

Negahdari E, Piraee K, Keshavarz Haddad G, Haghighat A. Economy of Scale and Poverty Line: A Case Study of Iranian Urban Households (2006- 2011). The Journal of Planning and Budgeting. 2014; 19 (1):3-30 <http://jpbud.ir/article-1-1134-fa.html>

____, Estimating Poverty Line of Iranian Rural Households with Respect to Household Size, 2006-2011. Quarterly Journal of Roosta Va Towse'e. 3. 2015; 17 (4):155-172 <http://rvt.agri-peri.ir:8080/browse.php?a_id=642&sid=1&slc_lang=en>

SCI (Statistical Center of Iran). “National Accounts: National Health Accounts of 1390”. Information extracted on August 05, 2015: <http://www.amar.org.ir/Default.aspx?tabid=104>

SSO (Social Security Organization, Iran). Information extracted from the following links on August 05, 2015. <http://www.tamin.ir/file/file/13896> , <http://en.tamin.ir/html/item/6><http://www.tamin.ir/News/Item/1806/2/1806.html>

1. Including inheritance tax and stamp duty. [↑](#footnote-ref-1)
2. Including real estate income tax, tax on income from agriculture, tax on salary income, tax on individual business income, tax on the profits of legal persons (i.e. Corporate income tax), incidental income tax and tax on aggregate income derived from different sources [↑](#footnote-ref-2)
3. A complete description of all taxable items is available (in English) from “Iranian National Tax Administration (INTA)” website: <http://en.intamedia.ir/> under the heading “Taxes in Iran”. [↑](#footnote-ref-3)
4. This is a simplified rule because some goods are not taxed and some (such as cigarettes) are taxed at higher rates. [↑](#footnote-ref-4)
5. Iranian household survey has income information for each household member for the year prior to the day of survey, but only expenditure information for the whole household for the month prior to that day. [↑](#footnote-ref-5)
6. In Farsi: “Tarh-e Hadafmansazi-e Yarane-ha”. [↑](#footnote-ref-6)
7. In Farsi: “Bonyad-e Shahid va Omoor-e Issargaran” [↑](#footnote-ref-7)
8. In Farsi: “Komite-ye Emdad-e Imam Khomeini” [↑](#footnote-ref-8)
9. In Farsi: “Bonyad-e Mostazafan-e Enghelab-e Eslami” [↑](#footnote-ref-9)
10. In Farsi: “Sazmane-e Behzisti-e Keshvar” [↑](#footnote-ref-10)
11. The total number of households in the extended survey is 21,159,033. [↑](#footnote-ref-11)
12. We define “Market Income” formally later in the paper but in a nutshell, it is equal to the factor income plus pensions minus contributions to pensions. [↑](#footnote-ref-12)
13. For example, a person who gets a four-year B.S. degree, depending on which public university he has attended, can be required to work for 12 years in the country before his degree is released to him/her. This requirement only affects those who wish to leave the country; for the rest of population, it is as if it did not exist. The only exception is for those who receive their degree from the Ministry of Health and Medical Education. They are required to work in public-run hospitals/medical centers of the government’s choice for a period of time upon graduation. [↑](#footnote-ref-13)
14. The imputed values for the in-kind education transfer for elementary, middle school, high school, and tertiary education are 4,462,000 , 6,431,000 , 9,853,666 , 58,800,000 Rials respectively. [↑](#footnote-ref-14)
15. We observe medical expenditure at the household level and allocate the health subsidy to all members of a family. [↑](#footnote-ref-15)
16. There are exceptions as to which employers are mandated to pay their share, or which employees are qualified for mandatory participation in SSO. Interested readers are encouraged to review the complete law (available on the SSO website). [↑](#footnote-ref-16)
17. The survey actually includes pre-tax income for employees. For the self-employed, market income is generated by subtracting Business Costs from Sales since there are questions for both items in the survey. [↑](#footnote-ref-17)
